# Circulating B Lymphocyte Subsets in Patients with Systemic Lupus Erythematosus

**DOI:** 10.3390/medicina60121994

**Published:** 2024-12-02

**Authors:** Joanna Kosałka-Węgiel, Bogdan Jakieła, Radosław Dziedzic, Mamert Milewski, Andżelika Siwiec-Koźlik, Lech Zaręba, Stanisława Bazan-Socha, Marek Sanak, Jacek Musiał, Mariusz Korkosz

**Affiliations:** 1 Jagiellonian University Medical College, Department of Rheumatology and Immunology, Jakubowskiego 2, 30-688 Kraków, Poland; mariusz.korkosz@uj.edu.pl; 2 University Hospital, Department of Rheumatology, Immunology and Internal Medicine, Jakubowskiego 2, 30-688 Kraków, Poland; mamert.mmm@gmail.com (M.M.); lek.andzelika.siwiec@gmail.com (A.S.-K.); stanislawa.bazan-socha@uj.edu.pl (S.B.-S.); 3 Jagiellonian University Medical College, Department of Internal Medicine, Faculty of Medicine, Jakubowskiego 2, 30-688 Kraków, Poland; b.jakiela@uj.edu.pl (B.J.); marek.sanak@uj.edu.pl (M.S.); jacek.musial@uj.edu.pl (J.M.); 4 Jagiellonian University Medical College, Doctoral School of Medical and Health Sciences, Św. Łazarza 16, 31-530 Kraków, Poland; radoslaw.dziedzic@doctoral.uj.edu.pl; 5 University of Rzeszów, College of Natural Sciences, Institute of Computer Science, Pigonia 1, 35-310 Rzeszów, Poland; lzareba@ur.edu.pl

**Keywords:** systemic lupus erythematosus, lupus nephritis, lymphocytes, CD19+ cells, B cells

## Abstract

*Background/Objectives*: Systemic lupus erythematosus (SLE) is an autoimmune disease characterized by the abnormal activation of autoreactive T and B cells, autoantibody production, complement activation, and immune-complex deposition, resulting in tissue damage. However, data on immunologic disturbances in SLE, particularly regarding flares, are scarce. *Methods*: We investigated 35 patients with SLE: 12 (34.3%) with disease exacerbation (SLE disease activity index [SLEDAI] ≥ 5 points) and 23 (65.7%) in remission (SLEDAI < 5 points). All patients met the 2019 EULAR/ACR SLE criteria. Flow cytometry was used to identify B cell subsets, including memory B cells. *Results*: In the whole patient group, SLEDAI was positively related to the percentage of transitional/regulatory B cells (r = 0.38, *p* = 0.034). Some lymphocyte subsets correlated with complement levels, e.g., the percentage of naïve and memory B cells showed associations with C3c complement (r = 0.43, *p* = 0.018 and r = −0.45, *p* = 0.016, respectively). Furthermore, regarding inflammatory markers, we found associations between C-reactive protein and the percentage of plasmablasts (r = 0.40, *p* = 0.026) and plasmocytes (r = 0.44, *p* = 0.017). Finally, the percentage of plasmablasts correlated with SLE duration (r = 0.42, *p* = 0.016). In the follow-up analysis, during a median observation of 5 years, 5 out of the initially 23 inactive SLE patients developed a disease flare. They were characterized by longer disease duration stated in the beginning compared to patients who remained in remission (*p* = 0.019). *Conclusions*: Our study highlights significant associations between various B cell subsets and SLE disease activity. A more personalized approach to indicate patients with SLE at a higher risk of lupus flares is crucial for better management.

## 1. Introduction

Systemic lupus erythematosus (SLE) is a multifaceted autoimmune disease characterized by the dysregulation of the immune system, leading to widespread inflammation and tissue damage across various organs [1]. This condition predominantly affects women, particularly those of childbearing age, and presents with a broad spectrum of clinical manifestations, ranging from mild symptoms such as fatigue and joint pain to severe organ involvement, including nephritis and central nervous system disorders [2,3]. The complexity of SLE stems from its underlying pathophysiology, which involves an aberrant activation of innate and adaptive immune responses [4]. Interestingly, a hallmark of SLE is the presence of autoantibodies that target a variety of nuclear and cytoplasmic antigens, resulting in the formation of immune complexes [5]. These complexes can deposit in tissues, activating the complement system and leading to inflammatory cascades that cause organ damage [6]. Central to the pathogenesis of SLE are autoreactive T and B cells, which play crucial roles in the sustained autoimmune response and production of pathogenic autoantibodies [7]. Despite significant advances in understanding the immunological disturbances in SLE, there are still gaps in knowledge regarding the specific immune profiles and biomarkers associated with disease flares [8]. Indeed, flares in SLE are episodes of increased disease activity that can significantly impact a patient’s quality of life and may lead to permanent organ damage if not adequately managed [9]. Identifying biomarkers and immune cell subsets that correlate with disease activity could enhance our ability to predict, monitor, and treat exacerbations effectively.

Emerging data indicate that B cell subsets may be essential for SLE pathophysiology, including exacerbation rate [10]. Plasmablasts and plasmocytes are involved in producing autoantibodies and regulating immune responses [11,12]. In turn, the B cell memory compartment in lupus nephritis (LN) is deficient in non-switched memory (NSM) cells. Next, during active disease, it becomes further skewed by the expansion of switched memory (SM) and exhausted memory phenotypes, most likely due to chronic antigenic stimulation [13]. In this context, regulatory B cells (B-regs) play a crucial role in modulating immune responses and maintaining tolerance. This is especially important in LN, where excessive immune activation can damage kidneys [14]. While interleukin (IL)-10 production is the most well-studied mechanism of B cell immune regulation, other IL-10-independent mechanisms have also been suggested [15]. Understanding how these lymphocyte subsets fluctuate during remission and exacerbation can provide valuable insights into the disease mechanism and potential therapeutic targets.

Evidence on immunologic disturbances in SLE regarding flares is still scarce. Therefore, in this study, we elucidated the changes within circulating B lymphocyte subsets in patients with SLE, particularly focusing on those experiencing disease exacerbation. Repeated flares can contribute to cumulative tissue damage over time, increasing the risk of long-term complications like chronic kidney disease and cardiovascular events [16]. By comparing the immune profiles of patients in remission and during flares, we hope to identify potentially useful immunologic biomarkers that correlate with disease activity and could serve as indicators for monitoring and managing SLE.

## 2. Patients and Methods

### 2.1. Characteristics of the Patients

This observational longitudinal study included 35 patients with SLE diagnosed according to the European League Against Rheumatism and the American College of Rheumatology (EULAR/ACR) criteria from 2019 [17]. We collected data about sex, present age, age at SLE onset, and disease duration. Blood samples were collected from all patients for B cell subset analysis.

The evaluation of LN was extended with a histologic type of nephropathy, classified according to the International Society of Nephrology/Renal Pathology Society (ISN/RPS) system (if kidney biopsy was performed) and numbers of LN exacerbations [18,19]. Renal biopsies were performed in 9 cases, representing 56.3% of LN patients. In 7 patients, renal biopsy was not performed due to lack of patient consent and/or medical contraindications. Patients with SLE were not previously treated with biological agents targeting B cells (e.g., rituximab, belimumab, and anifrolumab). We registered the current systemic glucocorticosteroids (GCS) dose and the cumulative cyclophosphamide (CTX) dose based on the clinical record. Importantly, patients were not treated with CTX 6 months preceding lymphocyte phenotyping. Disease activity was evaluated by the SLE disease activity index (SLEDAI) with renal SLEDAI (rSLEDAI) [20,21].

The Bioethics Committee of the Jagiellonian University Medical College approved the research (No: 1072.6120.34.2018, on 23 February 2018). All procedures adhered to the ethical principles outlined in the Declaration of Helsinki. Additionally, every individual received detailed information about the study’s methods and safety procedures and provided written informed consent to participate in the research.

### 2.2. Laboratory Analysis

We collected laboratory data, including hematological, renal, and immunological parameters. Complete blood count (CBC), C-reactive protein, creatinine with estimated glomerular filtration rate (eGFR, using Modification of Diet in Renal Disease formula) [22], 24 h urine protein excretion, and urinary sediment analysis were measured using routine laboratory techniques. Anti-nuclear antibodies (ANAs) were evaluated by indirect immunofluorescence (IIF). Anti-double-stranded DNA (anti-dsDNA) antibodies were assayed by IIF on *Critidia luciliae* and by enzyme-linked immunosorbent assay (ELISA, EUROIMMUN, Lübeck, Germany). Serum complement levels (C3c and C4) were assessed using laser nephelometry.

### 2.3. Flow Cytometry

**Figure 1:**
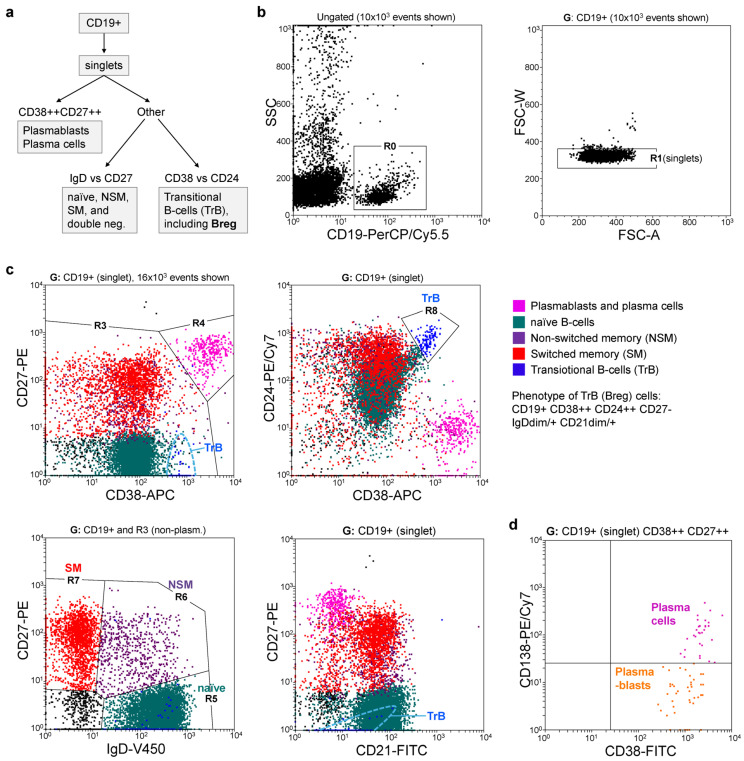
Flow cytometry staining of B cell subsets. (**a**) Schematic diagram showing the flow cytometry (FC) gating strategy used to identify major B cell subsets. Samples of peripheral blood mononuclear cells (PMBCs) were stained with pre-mixed antibodies (CD19-PerCP/Cy5.5, CD38-APC, CD27-PE, CD24-PE/Cy7, IgD-V450, CD21-FITC), and analyzed in FC (>10^3^ events in B cell gate recorded). (**b**,**c**) Representative dot plots showing gates and analysis flowchart. (**b**) CD19+ singlets (R0 + R1, only FSC-A/W gate shown) were first analyzed to distinguish the CD38++ CD27++ subset of plasma cells and plasmablasts (R4 region). (**c**) The remaining non-plasma cells were gated (R3) to identify naïve B- B cells, non-switched memory (NSM) B cells, and switched-memory (SM) B cells. Transitional B cells (TrB)/regulatory B cells (Breg) were identified as CD38++ CD24++ using an oblique gate (R8). TrB cells showed the following phenotype: CD19+ CD38++ CD24++ CD27− IgD(low/+) CD21(low/+). (**d**) An additional sample (stained with CD19-PerCP/Cy5.5, CD38-FITC, CD27-PE, CD138-PE/Cy7, IgD-V450, and IgM-APC) was used to distinguish between plasmablasts (CD138−) and plasma cells (CD138+). These two subsets were CD27++ and were negative for membrane IgD and IgM.

Aliquots of EDTA anticoagulated blood were stained with a mixture of monoclonal antibodies (all from BD Biosciences, Franklin Lakes, NJ, USA) for the detection of CD19+ B cells (anti-CD45, CD3, CD19, CD24, CD27, CD38, CD138, IgD; gating strategy shown in Figure 1). The samples were fixed with FACS lysing solution (BD Biosciences), washed, and analyzed in a FACS Canto II flow cytometer (BD Biosciences). Based on the differential expression of surface markers, the following B cell (CD19+) subsets were identified: transitional/regulatory (CD24+ CD38+) (B-trans/reg), naïve (IgD+ CD27−), non-switched memory (NSM, CD27+ IgD+), switched memory (SM, IgD− CD27+ CD38low), plasmablasts (CD38++ CD27++ CD24− CD138−), plasmocytes (CD38++ CD27++ CD24− CD138+), and double-negative B cells (IgD− CD27−) [13,23,24,25,26].

### 2.4. Statistical Elaboration

The results were analyzed using STATISTICA Tibco 13.3 software (StatSoft Inc., Tulsa, OK, USA). Categorical variables were presented as frequencies (number of cases) with relative frequencies (percentages) and compared using the Chi^2^ test or the exact Fisher test, as appropriate. The normality of data distribution was evaluated by the Shapiro–Wilk test. All continuous variables were non-normally distributed and, thus, were presented as medians with Q1–Q3 ranges and compared using the Mann–Whitney test. A Pearson correlation coefficient or a Spearman rank correlation test was used to analyze the associations between continuous variables. The level of statistical significance was α < 0.05.

## 3. Results

### 3.1. Clinical Characteristics

This study included 12 (34.3%) patients with SLE with disease exacerbation (SLEDAI ≥ 5 points) and 23 (65.7%) patients with SLE in remission (SLEDAI < 5 points). There was no difference in age, sex, age of disease onset, and duration between active and inactive SLE patients (Table 1).

Overall, the most frequent clinical SLE manifestations included hematological signs (n = 32, 91.4%), joint inflammation (n = 30, 85.7%), skin changes (n = 27, 77.1%), and photosensitivity (n = 16, 45.71%). In turn, serositis (n = 5, 14.3%), oral or nasopharyngeal ulcerations (n = 5, 14.3%), and nervous system involvement (n = 3, 8.6%) were much less common. Furthermore, all recruited patients had ANA titer ≥ 1:160, with anti-dsDNA antibodies being the most prevalent (n = 27, 77.1%). Regarding clinical parameters, we noticed higher SLEDAI and rSLEDAI scores with elevated ANA titer in the group of active SLE patients. Also, the active SLE patients had decreased concentrations of C3c and C4 complement components. Interestingly, at enrolment, we noticed more frequent renal manifestations and skin and joint involvement in the active group (Table 1).

LN was confirmed in 16 (45.1%) patients with SLE, of which in 5 (31.3%) cases, renal flare was diagnosed at the time of enrolment into the study. Overall, in LN patients, the most common histological type according to the ISN/RPS classification was class III, identified in 4 cases (44.4%), followed by classes IV (n = 3, 33.3%) and V (n = 2, 22.2%).

Only one patient was taking methylprednisolone > 8 mg/day (16 mg/day). Fourteen patients with SLE were previously (>6 months before blood sample) treated with CTX with a mean summary dose of 8.61 g. With the exception of hydroxychloroquine, none of the patients were taking other immunosuppressive drugs at the time of enrolment.

### 3.2. B Lymphocyte Subset Analysis in Active and Inactive Systemic Lupus Erythematosus Patients

Surprisingly, the analysis of B cell subset numbers and percentages revealed no significant differences between the active and inactive SLE groups (Table 2). In particular, the numbers and percentages of B-trans/reg cells were comparable between the analyzed subgroups (*p* > 0.05, both).

### 3.3. Detailed Characteristics of Active Systemic Lupus Erythematosus Patients

We divided active SLE patients into two subgroups, with renal flare (n = 5) and other than renal flare (n = 7), and compared them using clinical and laboratory parameters (Table 3). Demographic characteristics were similar in both subgroups. As expected, we noticed a higher prevalence of renal symptoms with higher SLEDAI and rSLEDAI scores in the renal flare subgroup. Nevertheless, there were no differences in the numbers and percentages of B lymphocyte subsets between the analyzed subgroups (Supplementary Table S1).

### 3.4. Detailed Characteristics of Non-Active Systemic Lupus Erythematosus Patients

Next, we divided remissive SLE patients into two subgroups: patients with renal remission (n = 10) and patients in remission with never-diagnosed LN (n = 13) and also compared clinical and laboratory parameters (Table 4). SLEDAI and rSLEDAI scores were comparable between both groups. Inactive systemic lupus erythematosus patients with remission and LN versus remission without LN also did not differ in numbers and percentages of B lymphocyte subsets (Supplementary Table S2).

### 3.5. B Cell Subsets Correlate with Systemic Lupus Erythematosus Activity

Interestingly, we observed several correlations between B cell subsets and selected SLE parameters. The percentage of trans/reg B cells showed a positive correlation with SLEDAI, ANAs titers, and C-reactive protein (CRP) levels (r = 0.38, r = 0.38, and r = 0.41, respectively; *p* < 0.05 for all). The percentage of naïve B cells was positively associated only with complement C3c component levels (r = 0.43, *p* = 0.018). Furthermore, the frequency of SM B cells correlated positively with the duration of SLE (r = 0.41, *p* = 0.028) and negatively with complement C3c concentrations (r = −0.45, *p* = 0.016). Plasmablast percentage was linked to SLE duration and CRP levels (r = 0.42 and r = 0.40, respectively; *p* < 0.05 for both). Similarly, the percentage of plasmocytes correlated with CRP levels (r = 0.44, *p* = 0.017). The proportion of DN B cells was associated with anti-dsDNA titers (r = 0.44, *p* = 0.02). Interestingly, the percentage of NSM B cells did not relate to the classical disease activity parameters. Detailed data are presented in below in Table 5.

### 3.6. Follow-Up Analysis of Inactive Systemic Lupus Erythematosus Patients

In the follow-up analysis, we carefully examined flares in inactive SLE patients from the enrolment over the median observation of 5 years. During this time, five patients developed a disease flare (Table 6). We also compared groups of inactive SLE patients with a flare and without a flare over this period to look for potential biomarkers. They had longer disease duration, and no differences in B cell subsets analyzed at the enrolment (Supplementary Table S3).

## 4. Discussion

This study explored the immunological profile of patients with SLE, particularly focusing on those experiencing disease exacerbations. Surprisingly, no significant differences were observed in the numbers or percentages of B cell subsets between patients with active and inactive SLE. However, correlations were identified regarding specific B cell subsets and disease activity parameters, providing valuable insights into the pathophysiology of SLE flares and highlighting potential immune-related markers for monitoring disease activity.

Importantly, the demographic profile of our cohort mirrored typical SLE populations, with a predominance of females (n = 29, 82.9%) [27]. There were no differences in age at study enrolment, age of SLE diagnosis, or disease duration between analyzed active and inactive SLE patients. However, despite similar demographics, distinct clinical and laboratory features emerged, underscoring the multifaceted nature of the disease. As expected, active SLE patients showed elevated levels of anti-dsDNA antibodies, more severe proteinuria, lower complement component levels (C3c and C4), and higher C-reactive protein levels than inactive SLE patients. These differences, similar to discrepancies in clinical symptoms, are typical of exacerbated SLE [28,29].

We did not find any significant differences in the numbers or percentages of B cell subsets in active and inactive SLE. This contrasts with our previous findings in lupus nephritis (LN) patients, where we observed an increased percentage of immature/early-transitional B cells (CD27^−^IgD^+^CD21^−^), a higher frequency of activated SM (CD27^+^IgD^−^CD21^−^) and exhausted memory B cells (CD27^−^IgD^−^), and a decrease in NSM (CD27^+^IgD^+^) B cells [13]. Our findings also differ from those presented by Rodríguez-Bayona et al. [30], who demonstrated a deficiency in SM B cells and an increased proportion of naive B cells in active SLE cases. The discrepancy between our results and existing data may be due to the small sample sizes of both studied groups.

On the other hand, in the entire cohort of 35 patients, disease activity, as measured by the SLEDAI, was positively associated with the percentage of trans/reg B cells, suggesting their engagement in the immunologic response of disease flare [31] and highlighting the significant role of the immune system in the progression of SLE.

Next, we also observed that specific B subsets were correlated with complement component levels. Complement components are clinically useful as they serve as biomarkers in SLE activity and are used in some SLE disease scores, such as SLEDAI [32]. Our observation suggests additional interplay between complement activation and lymphocyte dynamics in SLE. Indeed, the percentage of naïve and memory B cells was linked with C3c complement levels. C3, a central component of the complement system, plays a critical role in immune complex clearance and inflammatory responses [33]. Next, elevated levels of complement split products, such as C3dg, iC3b, C4d, and cell-bound complement activation products, can serve as diagnostic biomarkers and indicate disease activity, as well as predict adverse outcomes, including in antiphospholipid syndrome [33]. Dysfunctions in the complement system are pivotal in the pathogenesis of SLE, both during the initiation of autoimmunity and the inflammatory phase [32,33,34]. Complement deficiencies, such as C1q, are among the strongest genetic risk factors for SLE [32]. Complement activation on immune complexes and autoantibodies drives chronic inflammation, promoting the activation and expansion of autoreactive T and B cells. While complement receptor signaling on B cells typically helps regulate their activation, reduced receptor expression (e.g., CR2) in autoimmune diseases may impair this mechanism. Thus, targeting the inhibitory CR1 and CR2 in autoreactive B cells could serve as a novel scenario for therapeutic options in autoimmune diseases [34]. However, further investigation is needed to delineate which specific B cell subsets are primarily involved in this correlation and how they influence complement dynamics.

Another interesting finding of our study is a positive correlation between an unspecific marker of inflammation CRP and the percentage of plasmablasts and plasmocytes, underscoring the role of these cells in the SLE flares. Plasmablasts and plasmocytes produce autoantibodies, which form immune complexes that deposit in tissues and activate the complement system, perpetuating the cycle of inflammation [35], especially in the kidneys, skin, and joints [36]. In turn, elevated CRP points to the active inflammation [37]. Thus, the association presented here reinforces these cells as central to the humoral immune response and simultaneously to the acute inflammatory response in SLE. Notably, the percentage of plasmablasts also showed a significant correlation with the duration of SLE, which may reflect the chronic activation and differentiation of B cells over the disease course.

One of the crucial aspects of SLE management is the presence of LN [38]. However, there are no reliable early non-invasive biomarkers for ongoing kidney damage [39]. For example, Kitagori et al. [40] proposed a urine osteopontin N-half concentration as a novel biomarker related to kidney inflammation; however, more extensive clinical studies are needed to confirm its suitability in LN prediction. Furthermore, previously, it was documented that some B cell compartments were deficient in those cases [13]. We did not find such an association; only a positive correlation was noted between CD19+ cells and proteinuria. The lack of significant correlations between CD19+ cells and other kidney function markers, such as creatinine or eGFR, suggests proteinuria, a hallmark feature of lupus nephritis [41], might be a more sensitive marker of immune-mediated kidney damage in SLE. Importantly, ongoing proteinuria is a risk factor for LN flares in patients discontinuing immunosuppressive therapy [42]. Therefore, monitoring CD19+ B cell levels, together with proteinuria, might serve as a specific marker for assessing the risk and severity of renal involvement in patients with SLE. This could be especially valuable in clinical settings where early detection of kidney involvement is crucial for preventing progression to more severe renal damage and chronic kidney disease. Additionally, targeting B cells with therapies such as B cell depletion (e.g., rituximab) might be particularly effective for patients with SLE with significant renal involvement, as it directly addresses a key component of the pathogenic mechanism leading to proteinuria [43]. Furthermore, similar to others [44,45,46], we showed that specific immunological profiles could be linked with disease activity referring to the SLEDAI score.

A novelty of our study was the long-term follow-up of inactive SLE patients to identify immunological markers of pre-active or smoldering disease. However, a few of those with exacerbations (n = 5, 21.7% out of 23 inactive SLE patients) do not allow us to draw reliable conclusions. In contrast, other researchers underscore that specific B cell subpopulations may be valuable biomarkers for flare prediction [47].

## 5. Limitations of the Study

Despite its valuable findings, this study has several limitations. Firstly, the relatively small sample size limits the statistical power and may affect the reliability of the conclusions. Secondly, the single-center design introduces the possibility of selection bias, which may reduce the generalizability of the findings to broader populations. Furthermore, the cross-sectional nature of the study restricts the ability to establish causal relationships, particularly in exploring predictive factors for relapses or disease progression. Additionally, while informative, the study overlooks other potential aspects of disease mechanisms. Future approaches will focus on cytokine profiles, genetic markers, and activation markers on B cells to better understand disease mechanisms and identify potential therapeutic targets.

## 6. Conclusions

Our study highlights significant associations between various B cell subsets and SLE disease activity. Some B lymphocyte subsets correlated with complement levels support its role as a biomarker for identifying and tracking exacerbations in patients with SLE. These findings contribute to a better understanding of disease variability and could inform clinical management strategies for patients with SLE, especially those with renal manifestations. Future studies with larger, multicenter cohorts and prospective designs are needed to validate our findings and elucidate the long-term impact of identified markers on clinical outcomes in SLE. A more personalized approach to indicate patients with SLE at a higher risk of lupus flares is crucial for better management.

## Figures and Tables

**Table 1:** Demographic and clinical characteristics of active and inactive systemic lupus erythematosus patients.

Parameter	Active SLE Patients n = 12	Inactive SLE Patients n = 23	*p*-Value
**Demographic characteristics**			
Age at study enrolment, years	46.0 (36.5–50.5)	59.0 (41.0–65.0)	0.07
Sex, female, n (%)	11 (91.7%)	18 (78.3%)	0.64
Age at the SLE onset, years	24.0 (18.3–34.8)	31.0 (22.0–39.0)	0.26
SLE duration, years	16.5 (6.0–25.8)	20.0 (15.0–26.0)	0.26
**Symptoms ever stated**			
Skin, n (%)	9 (75.0%)	18 (78.3%)	1.00
Oral or nasopharyngeal ulcerations, n (%)	1 (8.3%)	4 (17.3%)	0.64
Photosensitivity, n (%)	7 (58.3%)	9 (39.1%)	0.28
Joints, n (%)	10 (83.3%)	20 (87.0%)	1.00
Serositis, n (%)	3 (25.0%)	2 (8.7%)	0.31
Renal, n (%)	6 (50.0%)	10 (43.5%)	0.71
Neurologic, n (%)	0 (0.0%)	3 (13.0%)	0.54
Hematologic, n (%)	11 (91.7%)	21 (91.3%)	1.00
**Symptoms at the study enrolment**			
Skin, n (%)	5 (41.7%)	2 (8.7%)	**0.033**
Joints, n (%)	6 (50.0%)	0 (0.0%)	**<0.001**
Serositis, n (%)	1 (8.3%)	0 (0.0%)	0.34
Renal, n (%)	5 (41.7%)	0 (0.0%)	**0.002**
Neurologic, n (%)	0 (0.0%)	0 (0.0%)	NA
Hematologic, n (%)	6 (50.0%)	4 (17.3%)	0.06
**Clinical characteristics**			
SLEDAI, score	12.0 (8.5–15.5)	2.0 (0.0–2.0)	**<0.001**
rSLEDAI, score	2.0 (0.0–12.0)	0.0 (0.0–0.0)	**0.016**
ANAs, titer	1:20,480 (1:2560–1:20,480)	1:2560 (1:160–1:20,480)	**0.002**
Anti-dsDNA antibodies, titer	1:80 (<1:10–1:5120)	<1:10 (<1:10–1:320)	**0.037**
Proteinuria, g/day	0.41 (0.10–1.66)	0.11 (0.08–0.14)	**0.007**
Creatinine, µmol/L	58.5 (51.0–89.0)	61.0 (56.0–83.0)	0.69
eGFR, mL/min/1.73 m^2^	103.5 (65.3–121.5)	97.0 (82.0–107.0)	0.42
C3c, mg/dL	0.77 (0.51–1.01)	0.96 (0.82–1.20)	**0.013**
C4, mg/dL	0.08 (0.07–0.16)	0.17 (0.13–0.21)	**0.003**
C-reactive protein, mg/L	3.0 (1.4–16.0)	1.2 (1.0–3.3)	0.06
White blood cells, 10^3^/µL	4.9 (3.2–6.9)	5.5 (3.6–8.8)	0.29
Lymphocytes, 10^3^/µL	1.0 (0.5–1.6)	1.2 (0.9–1.7)	0.23
Neutrophils, 10^3^/µL	3.3 (1.9–5.3)	4.1 (2.3–6.6)	0.46
Platelets, 10^3^/µL	241 (145–280)	246 (214–296)	0.40
Current systemic GCS, mg/day	0.0 (0.0–5.5)	0.0 (0.0–4.0)	0.55
Cumulative CTX dose, g	4.0 (0.0–26.5)	0.0 (0.0–9.0)	0.38

Categorical variables are presented as numbers with percentages. Continuous variables as median with Q1–Q3 ranges (medians with min-max ranges in case of ANAs and anti-dsDNA titers). Statistically significant differences are marked in bold. Abbreviations: ANAs—anti-nuclear antibodies, C3c—complement component 3c, C4—complement component 4, CTX—cyclophosphamide, eGFR—estimated Glomerular Filtration Rate, GCS—glucocorticosteroids (recounted for methylprednisolone), NA—not applicable, n—number, rSLEDAI—renal SLEDAI, SLE—systemic lupus erythematosus, SLEDAI—SLE disease activity index.

**Table 2:** B lymphocyte subset analysis in active and inactive systemic lupus erythematosus patients.

Parameter	Active SLE Patients n = 12	Inactive SLE Patients n = 23	*p*-Value
CD19+, % of lymphocytes	7.9 (5.6–17.5)	5.6 (3.6–10.0)	0.09
Naive, % of B lymphocytes	60.5 (45.1–77.3)	72.7 (53.9–84.7)	0.37
SM, % of B lymphocytes	14.1 (6.2–31.4)	13.4 (5.9–24.6)	0.87
NSM, % of B lymphocytes	8.7 (3.3–10.4)	7.1 (3.4–10.5)	0.87
DN, % of B lymphocytes	5.0 (1.4–9.6)	2.7 (1.4–5.3)	0.46
Plasmocytes, % of B lymphocytes	0.6 (0.1–1.7)	0.4 (0.2–0.7)	0.70
Plasmablasts, % of B lymphocytes	1.3 (0.5–3.7)	1.2 (0.5–3.3)	0.73
B-trans/reg, % of B lymphocytes	3.8 (2.1–5.0)	2.2 (0.7–3.6)	0.10
B cells per μL			
CD19+, cells/μL	91.6 (35.9–176.4)	88.5 (41.6–145.0)	0.87
Naive, cells/μL	39.9 (14.6–126.4)	53.2 (17.3–120.3)	0.79
SM, cells/μL	11.7 (7.4–26.9)	11.6 (5.2–18.2)	0.71
NSM, cells/μL	6.1 (3.0–8.3)	5.7 (1.8–9.3)	0.68
DN, cells/μL	3.5 (1.6–4.8)	2.2 (0.8–3.7)	0.24
Plasmocytes, cells/μL	0.5 (0.2–0.8)	0.2 (0.1–0.6)	0.29
Plasmablasts, cells/μL	1.3 (0.4–2.0)	0.7 (0.4–2.0)	0.55
B-trans/reg, cells/μL	3.8 (0.9–5.2)	1.7 (0.3–4.9)	0.40

Continuous variables are presented as medians with Q1–Q3 ranges. Abbreviations: B trans/reg—transitional/regulatory B cells, CD—cluster of differentiation, DN—double negative, n—number, NSM—non-switched memory, SLE—systemic lupus erythematosus, SM—switched memory.

**Table 3:** Demographic and clinical characteristics of active systemic lupus erythematosus patients with renal flare and other than renal flare.

Parameter	Active SLE Patients with Renal Flare n = 5	Active SLE Patients Other Than Renal Flare n = 7	*p*-Value
**Demographic characteristics**			
Age, years	42.0 (30.0–50.0)	48.0 (36.0–59.0)	0.53
Sex, female, n (%)	5 (100.0%)	6 (85.7%)	1.00
Age at the SLE onset, years	26.0 (19.5–32.5)	22.0 (18.0–44.0)	0.88
SLE duration, years	17.0 (6.0–21.5)	16.0 (5.0–36.0)	0.76
**Symptoms ever stated**			
Skin, n (%)	3 (60.0%)	6 (85.7%)	0.52
Oral or nasopharyngeal ulcerations, n (%)	0 (0.0%)	1 (14.3%)	1.00
Photosensitivity, n (%)	3 (60.0%)	4 (57.1%)	1.00
Joints, n (%)	3 (60.0%)	7 (100.0%)	0.15
Serositis, n (%)	2 (40.0%)	1 (14.3%)	0.52
Renal, n (%)	5 (100.0%)	1 (14.3%)	**0.015**
Neurologic, n (%)	0 (0.0%)	0 (0.0%)	NA
Hematologic, n (%)	4 (80.0%)	7 (100.0%)	0.42
**Symptoms at the study enrolment**			
Skin, n (%)	1 (20.0%)	4 (57.1%)	0.29
Joints, n (%)	1 (20.0%)	5 (71.4%)	0.24
Serositis, n (%)	1 (20.0%)	0 (0.0%)	0.42
Renal, n (%)	5 (100.0%)	0 (0.0%)	**0.001**
Neurologic, n (%)	0 (0.0%)	0 (0.0%)	NA
Hematologic, n (%)	4 (80.0%)	2 (28.6%)	0.24
**Clinical characteristics**			
SLEDAI, score	16.0 (12.5–22.5)	10.0 (6.0–12.0)	**0.010**
rSLEDAI, score	12.0 (10.0–14.0)	0.0 (0.0–0.0)	**0.003**
ANAs, titer	1:5120 (1:2560–1:20,480)	1:20,480 (1:2560–1:20,480)	0.11
Anti-dsDNA antibodies, titer	1:1280 (<1:10–1:5120)	1:20 (<1:10–1:5120)	0.43
Proteinuria, g/day	1.79 (1.10–7.32)	0.14 (0.08–0.34)	**0.003**
Creatinine, µmol/L	93.0 (47.5–206.5)	57.0 (54.0–72.0)	0.43
eGFR, mL/min/1.73 m^2^	59.0 (26.0–134.5)	105.0 (100.0–111.0)	0.53
C3c, mg/dL	0.80 (0.42–1.02)	0.74 (0.50–1.06)	0.88
C4, mg/dL	0.11 (0.06–0.17)	0.08 (0.07–0.17)	0.88
C-reactive protein, mg/L	2.1 (1.1–26.1)	5.2 (2.2–18.8)	0.53
White blood cells, 10^3^/µL	4.7 (2.6–7.9)	5.0 (4.4–6.3)	0.76
Lymphocytes, 10^3^/µL	0.9 (0.6–2.1)	1.0 (0.4–1.6)	0.88
Neutrophils, 10^3^/µl	3.1 (1.4–5.8)	3.4 (2.3–5.3)	0.53
Platelets, 10^3^/µL	242 (193–297)	204 (129–260)	0.43
Current systemic GCS, mg/day	0.0 (0.0–2.0)	4.0 (0.0–8.0)	0.20
Cumulative CTX dose, g	10.0 (0.0–35.8)	0.0 (0.0–10.0)	0.43

Categorical variables are presented as numbers with percentages. Continuous variables as medians with Q1–Q3 ranges (medians with min-max ranges in ANAs and anti-dsDNA titers). Statistically significant differences are marked in bold. Abbreviations: ANAs—anti-nuclear antibodies, C3c—complement component 3c, C4—complement component 4, CTX—cyclophosphamide, eGFR—estimated Glomerular Filtration Rate, GCS—glucocorticosteroids (recounted for methylprednisolone), NA—not applicable, n—number, rSLEDAI—renal SLEDAI, SLE—systemic lupus erythematosus, SLEDAI—SLE disease activity index.

**Table 4:** Demographic and clinical characteristics of inactive systemic lupus erythematosus patients with remission and lupus nephritis versus remission without lupus nephritis.

Parameter	Inactive SLE Patients with Remission and Lupus Nephritis n = 10	Inactive SLE Patients with Remission Without Lupus Nephritis n = 13	*p*-Value
**Demographic characteristics**			
Age, years	44.5 (36.8–66.0)	61.0 (49.5–66.0)	0.19
Sex, female, n (%)	7 (70.0%)	11 (84.6%)	0.62
Age at the SLE onset, years	24.5 (20.0–32.5)	36.0 (22.5–42.0)	0.26
SLE duration, years	16.5 (9.3–28.0)	23.0 (18.0–33.0)	0.23
**Symptoms ever stated**			
Skin, n (%)	7 (70.0%)	11 (84.6%)	0.62
Oral or nasopharyngeal ulcerations, n (%)	2 (20.0%)	2 (15.4%)	1.00
Photosensitivity, n (%)	4 (40.0%)	5 (38.3%)	1.00
Joints. n (%)	9 (90.0%)	11 (84.6%)	1.00
Serositis, n (%)	1 (10.0%)	1 (7.7%)	1.00
Renal, n (%)	10 (100.0%)	0 (0.0%)	**<0.001**
Neurologic, n (%)	2 (20.0%)	1 (7.7%)	0.56
Hematologic, n (%)	10 (100.0%)	11 (84.6%)	0.49
**Symptoms at the study enrolment**			
Skin, n (%)	1 (10.0%)	1 (7.7%)	1.00
Joints, n (%)	0 (0.0%)	0 (0.0%)	NA
Serositis, n (%)	0 (0.0%)	0 (0.0%)	NA
Renal, n (%)	0 (0.0%)	0 (0.0%)	NA
Neurologic, n (%)	0 (0.0%)	0 (0.0%)	NA
Hematologic, n (%)	1 (10.0%)	3 (23.1%)	0.60
**Clinical characteristics**			
SLEDAI, score	1.0 (0.0–2.5)	2.0 (1.0–3.0)	0.38
rSLEDAI, score	0.0 (0.0–0.0)	0.0 (0.0–0.0)	1.00
ANAs, titer	1:1280 (1:160–1:10,240)	1:5120 (1:320–1:20,480)	**0.042**
Anti-dsDNA antibodies, titer	<1:10 (<1:10–1:320)	<1:10 (<1:10–1:160)	0.93
Proteinuria, g/day	0.13 (0.08–0.18)	0.10 (0.08–0.12)	0.26
Creatinine, µmol/L	70.0 (58.3–91.8)	59.0 (53.5–72.0)	0.10
eGFR, mL/min/1.73 m^2^	82.5 (71.3–103.0)	99.0 (86.0–108.5)	0.21
C3c, mg/dL	1.09 (0.89–1.36)	0.90 (0.81–1.12)	0.17
C4, mg/dL	0.19 (0.14–0.22)	0.17 (0.11–0.21)	0.34
C-reactive protein, mg/L	2.6 (1.2–4.7)	1.0 (1.0–2.4)	0.11
White blood cells, 10^3^/µL	7.7 (4.8–9.5)	4.6 (3.4–8.0)	**0.042**
Lymphocytes, 10^3^/µL	1.7 (1.4–2.0)	1.0 (0.7–1.2)	**0.003**
Neutrophils, 10^3^/µL	4.1 (2.5–6.8)	3.8 (2.1–6.0)	0.28
Platelets, 10^3^/µL	280 (180–336)	243 (220–264)	0.38
Current systemic GCS, mg/day	0.0 (0.0–4.0)	0.0 (0.0–3.0)	0.83
Cumulative CTX dose, g	7.5 (0.0–33.2)	0.0 (0.0–0.0)	**0.010**

Categorical variables are presented as numbers with percentages. Continuous variables as medians with Q1–Q3 ranges (medians with min-max ranges in ANAs and anti-dsDNA titers). Statistically significant differences are marked in bold. Abbreviations: ANAs—anti-nuclear antibodies, C3c—complement component 3c, C4—complement component 4, CTX—cyclophosphamide, GCS—glucocorticosteroids (recounted for methylprednisolone), eGFR—estimated Glomerular Filtration Rate, NA—not applicable, n—number, rSLEDAI—renal SLEDAI, SLE—systemic lupus erythematosus, SLEDAI—SLE disease activity index.

**Table 5:** Association of B cell subsets with clinical and laboratory parameters of systemic lupus erythematosus.

Parameter	CD19+, Cells/μL	Transitional/Regulatory, % of B Lymphocytes	Naïve, % of B Lymphocytes	NSM, % of B Lymphocytes	SM, % of B Lymphocytes	Plasmablasts, % of B Lymphocytes	Plasmocytes, % of B Lymphocytes	DN, % of B Lymphocytes
SLEDAI, points	r = 0.19, *p* = 0.28	**r = 0.38,** ***p* = 0.034**	r = −0.12, *p* = 0.51	r = 0.13, *p* = 0.51	r = 0.02, *p* = 0.93	r = 0.10, *p* = 0.59	r = 0.11, *p* = 0.56	r = 0.19, *p* = 0.35
rSLEDAI, points	r = 0.24, *p* = 0.17	r = 0.28, *p* = 0.12	r = −0.14, *p* = 0.43	r = 0.22, *p* = 0.27	r = 0.10, *p* = 0.60	r = −0.12, *p* = 0.52	r = 0.09, *p* = 0.64	r = 0.06, *p* = 0.75
Anti-dsDNA, titer	r = −0.05, *p* = 0.77	r = 0.33, *p* = 0.07	r = −0.02, *p* = 0.92	r = −0.11, *p* = 0.58	r = −0.19, *p* = 0.33	r = 0.15, *p* = 0.41	r = 0.14, *p* = 0.46	**r = 0.44,** ***p* = 0.020**
ANAs, titer	r = 0.03, *p* = 0.85	**r = 0.38,** ***p* = 0.035**	r = 0.10, *p* = 0.59	r = 0.14, *p* = 0.50	r = −0.06, *p* = 0.75	r = 0.13, *p* = 0.47	r = 0.25, *p* = 0.19	r = 0.37, *p* = 0.06
C-reactive protein, mg/L	r = −0.19, *p* = 0.28	**r = 0.41,** ***p* = 0.023**	r = 0.00, *p* = 0.98	r = −0.21, *p* = 0.29	r = −0.08, *p* = 0.70	**r = 0.40,** ***p* = 0.026**	**r = 0.44,** ***p* = 0.017**	r = 0.13, *p* = 0.53
Creatinine, µmol/L	r = 0.16, *p* = 0.36	r = −0.14, *p* = 0.44	r = −0.15, *p* = 0.41	r = −0.21, *p* = 0.29	r = −0.11, *p* = 0.58	r = −0.17, *p* = 0.37	r = −0.16, *p* = 0.40	r = −0.26, *p* = 0.18
eGFR, mL/min/1.73 m^2^	r = −0.10, *p* = 0.58	r = 0.10, *p* = 0.58	r = 0.08, *p* = 0.66	r = 0.34, *p* = 0.08	r = 0.09, *p* = 0.66	r = 0.21, *p* = 0.25	r = 0.23, *p* = 0.23	r = 0.26, *p* = 0.20
C3c, mg/dL	r = 0.22, *p* = 0.21	r = −0.19, *p* = 0.30	**r = 0.43,** ***p* = 0.018**	r = −0.33, *p* = 0.09	**r = −0.45,** ***p* = 0.016**	r = −0.05, *p* = 0.79	r = −0.31, *p* = 0.09	r = −0.28, *p* = 0.15
C4, mg/dL	r = 0.16, *p* = 0.35	r = −0.19, *p* = 0.30	r = 0.28, *p* = 0.12	r = −0.25, *p* = 0.19	r = −0.30, *p* = 0.12	r = −0.02, *p* = 0.90	r = 0.11, *p* = 0.55	r = −0.18, *p* = 0.36
SLE duration, years	**r = −0.38,** ***p* = 0.026**	r = 0.17, *p* = 0.35	r = −0.12, *p* = 0.51	r = 0.25, *p* = 0.21	**r = 0.41,** ***p* = 0.028**	**r = 0.42,** ***p* = 0.016**	r = 0.31, *p* = 0.09	r = 0.17, *p* = 0.38
Proteinuria, g/day	**r = 0.43,** ***p* = 0.011**	r = 0.15, *p* = 0.42	r = −0.08, *p* = 0.67	r = 0.18, *p* = 0.37	r = −0.18, *p* = 0.35	r = −0.09, *p* = 0.64	r = −0.02, *p* = 0.91	r = 0.21, *p* = 0.28
Cumulative CTX dose, g	r = 0.01, *p* = 0.97	r = −0.07, *p* = 0.71	r = −0.11, *p* = 0.56	r = −0.16, *p* = 0.42	r = 0.18, *p* = 0.36	r = −0.05, *p* = 0.79	r = −0.13, *p* = 0.48	r = 0.23, *p* = 0.24

Correlation matrix showing significant associations between the main subsets of B cells and selected markers of active systemic lupus erythematosus (in the whole SLE group). Statistically significant differences are marked in bold. Abbreviations: ANAs—antinuclear antibodies, B trans/reg—transitional/regulatory B cells, C3c—complement component 3c, C4—complement component 4, CD—cluster of differentiation, CTX—cyclophosphamide (cumulative dose), eGFR—estimated Glomerular Filtration Rate, DN—double negative, n—number, NSM—non-switched memory, SLE—systemic lupus erythematosus, SM—switched memory, rSLEDAI—renal SLEDAI, SLE—systemic lupus erythematosus, SLEDAI—SLE disease activity index.

**Table 6:** Demographic and clinical characteristics of inactive systemic lupus erythematosus patients with a flare and without a flare in the follow-up analysis.

Parameter	Inactive SLE Patients with a Flare in the Follow-Up n = 5	Inactive SLE Patients Without a Flare in the Follow-Up n = 18	*p*-Value
**Demographic characteristics**			
Age, years	63.0 (52.0–69.0)	52.5 (38.5–65.0)	0.20
Sex, female, n (%)	4 (80.0%)	14 (77.8%)	1.00
Age at the SLE onset, years	23.0 (22.5–37.5)	31.0 (20.0–41.5)	0.80
SLE duration, years	25.0 (22.0–46.5)	18.0 (11.0–23.8)	**0.019**
**Symptoms ever stated**			
Skin, n (%)	4 (80.0%)	14 (77.8%)	1.00
Oral or nasopharyngeal ulcerations, n (%)	2 (40.0%)	2 (11.1%)	0.19
Photosensitivity, n (%)	3 (60.0%)	6 (33.3%)	0.34
Joints, n (%)	5 (100.0%)	15 (83.3%)	1.00
Serositis, n (%)	0 (0.0%)	2 (11.1%)	1.00
Renal, n (%)	2 (40.0%)	8 (44.4%)	1.00
Neurologic, n (%)	1 (20.0%)	2 (11.1%)	0.54
Hematologic, n (%)	5 (100.0%)	16 (88.9%)	1.00
**Symptoms at the study enrolment**			
Skin, n (%)	0 (0.0%)	2 (11.1%)	1.00
Joints, n (%)	0 (0.0%)	0 (0.0%)	NA
Serositis, n (%)	0 (0.0%)	0 (0.0%)	NA
Renal, n (%)	0 (0.0%)	0 (0.0%)	NA
Neurologic, n (%)	0 (0.0%)	0 (0.0%)	NA
Hematologic, n (%)	0 (0.0%)	4 (22.2%)	0.54
**Clinical characteristics**			
SLEDAI, score	2.0 (1.0–2.0)	2.0 (0.0–4.0)	0.91
rSLEDAI, score	0.0 (0.0–0.0)	0.0 (0.0–0.0)	1.00
ANAs, titer	1:10,240 (1:1280–1:20,480)	1:1280 (1:160–1:20,480)	0.08
Anti-dsDNA antibodies, titer	<1:10 (<1:10–1:80)	<1:10 (<1:10–1:320)	0.36
Proteinuria, g/day	0.11 (0.05–0.16)	0.11 (0.08–0.14)	0.86
Creatinine, µmol/L	65.0 (59.0–92.0)	60.5 (52.5–80.8)	0.36
eGFR, mL/min/1.73 m^2^	83.0 (66.0–99.0)	97.5 (82.0–111.0)	0.26
C3c, mg/dL	0.86 (0.66–1.16)	0.99 (0.82–1.32)	0.49
C4, mg/dL	0.17 (0.11–0.23)	0.18 (0.13–0.22)	0.86
C-reactive protein, mg/L	2.6 (1.3–3.1)	1.0 (1.0–3.7)	0.40
White blood cells, 10^3^/µL	3.6 (3.0–7.3)	6.4 (4.4–8.9)	0.23
Lymphocytes, 10^3^/µL	0.9 (0.7–2.5)	1.3 (1.0–1.7)	0.23
Neutrophils, 10^3^/µL	2.8 (1.7–4.1)	4.6 (2.3–6.7)	0.20
Platelets, 10^3^/µL	262 (162–312)	245 (217–273)	1.00
Current systemic GCS, mg/day	0.0 (0.0–4.0)	0.0 (0.0–2.5)	0.86
Cumulative CTX dose, g	0.0 (0.0–22.1)	0.0 (0.0–5.5)	0.91

Categorical variables are presented as numbers with percentages. Continuous variables as medians with Q1–Q3 ranges (medians with a min-max range in case of ANAs and anti-dsDNA titers). Statistically significant differences are marked in bold. Abbreviations: ANAs—anti-nuclear antibodies, C3c—complement component C3c, C4—complement component 4, CTX—cyclophosphamide, eGFR—estimated Glomerular Filtration Rate, GCS—glucocorticosteroids (recounted for methylprednisolone), NA—not applicable, n—number, rSLEDAI—renal SLEDAI, SLE—systemic lupus erythematosus, SLEDAI—SLE disease activity index.

## Data Availability

The data presented in this study are available upon reasonable request from the corresponding author.

## Supplementary Materials

The following supporting information can be downloaded at https://www.mdpi.com/article/10.3390/medicina60121994/s1. Table S1: B cell subset analysis in active systemic lupus erythematosus patients with renal flare and other than renal flare; Table S2: B cell subset analysis in active systemic lupus erythematosus patients with remission and lupus nephritis versus remission without lupus nephritis; Table S3: B cell subset analysis in inactive systemic lupus erythematosus patients with a flare and without a flare in the follow-up analysis.

## References

[1] Hoi A., Igel T., Mok C.C., Arnaud L. (2024). Systemic Lupus Erythematosus. Lancet.

[2] Lisnevskaia L., Murphy G., Isenberg D. (2014). Systemic Lupus Erythematosus. Lancet.

[3] Piga M., Tselios K., Viveiros L., Chessa E., Neves A., Urowitz M.B., Isenberg D. (2024). Clinical Patterns of Disease: From Early Systemic Lupus Erythematosus to Late-Onset Disease. Best Pract. Res. Clin. Rheumatol..

[4] Hisada R., Kono M. (2024). Potential Therapies Targeting Metabolic Pathways in Systemic Lupus Erythematosus. Clin. Immunol..

[5] Dema B., Charles N. (2016). Autoantibodies in SLE: Specificities, Isotypes and Receptors. Antibodies.

[6] Accapezzato D., Caccavale R., Paroli M.P., Gioia C., Nguyen B.L., Spadea L., Paroli M. (2023). Advances in the Pathogenesis and Treatment of Systemic Lupus Erythematosus. Int. J. Mol. Sci..

[7] D’Cruz D.P., Khamashta M.A., Hughes G.R. (2007). Systemic Lupus Erythematosus. Lancet.

[8] Yu H., Nagafuchi Y., Fujio K. (2021). Clinical and Immunological Biomarkers for Systemic Lupus Erythematosus. Biomolecules.

[9] Moysidou G.-S., Mastrogiorgakis D., Boumpas D., Bertsias G. (2023). Management of Systemic Lupus Erythematosus: A New Scenario. Best Pract. Res. Clin. Rheumatol..

[10] Morand E.F., Fernandez-Ruiz R., Blazer A., Niewold T.B. (2023). Advances in the Management of Systemic Lupus Erythematosus. BMJ.

[11] Fujio K., Okamura T., Sumitomo S., Yamamoto K. (2013). Regulatory Cell Subsets in the Control of Autoantibody Production Related to Systemic Autoimmunity. Ann. Rheum. Dis..

[12] Nutt S.L., Hodgkin P.D., Tarlinton D.M., Corcoran L.M. (2015). The Generation of Antibody-Secreting Plasma Cells. Nat. Rev. Immunol..

[13] Kosalka J., Jakiela B., Musial J. (2016). Changes of Memory B- and T-Cell Subsets in Lupus Nephritis Patients. Folia Histochem. Cytobiol..

[14] Bilate A.M., Lafaille J.J. (2012). Induced CD4^+^Foxp3^+^ Regulatory T Cells in Immune Tolerance. Annu. Rev. Immunol..

[15] Ray A., Wang L., Dittel B.N. (2015). IL-10-Independent Regulatory B-Cell Subsets and Mechanisms of Action. Int. Immunol..

[16] Lam N.-C.V., Brown J.A., Sharma R. (2023). Systemic Lupus Erythematosus: Diagnosis and Treatment. Am. Fam. Physician.

[17] Aringer M. (2019). EULAR/ACR Classification Criteria for SLE. Semin. Arthritis Rheum..

[18] Kaczmarczyk K., Kosalka J., Soja J., Kuzniewski M., Musial J., Okon K. (2014). Renal Interstitial Mast Cell Counts Differ across Classes of Proliferative Lupus Nephritis. Folia Histochem. Cytobiol..

[19] Szymanik-Grzelak H., Barabasz M., Wikiera-Magott I., Banaszak B., Wieczorkiewicz-Płaza A., Bieniaś B., Drożynska-Duklas M., Tkaczyk M., Pańczyk-Tomaszewska M. (2021). Retrospective Analysis of Clinical and Pathomorphological Features of Lupus Nephritis in Children. Adv. Med. Sci..

[20] Jakiela B., Kosałka J., Plutecka H., Węgrzyn A.S., Bazan-Socha S., Sanak M., Musiał J. (2018). Urinary Cytokines and mRNA Expression as Biomarkers of Disease Activity in Lupus Nephritis. Lupus.

[21] Aljaberi N., Wenderfer S.E., Mathur A., Qiu T., Jose S., Merritt A., Rose J., Devarajan P., Huang B., Brunner H. (2022). Clinical Measurement of Lupus Nephritis Activity Is Inferior to Biomarker-Based Activity Assessment Using the Renal Activity Index for Lupus Nephritis in Childhood-Onset Systemic Lupus Erythematosus. Lupus Sci. Med..

[22] Levey A.S., Titan S.M., Powe N.R., Coresh J., Inker L.A. (2020). Kidney Disease, Race, and GFR Estimation. CJASN.

[23] Żabińska M., Kościelska-Kasprzak K., Krajewska J., Bartoszek D., Augustyniak-Bartosik H., Krajewska M. (2021). Immune Cells Profiling in ANCA-Associated Vasculitis Patients—Relation to Disease Activity. Cells.

[24] Chung M.K.Y., Gong L., Kwong D.L., Lee V.H., Lee A.W., Guan X., Kam N., Dai W. (2023). Functions of Double-negative B Cells in Autoimmune Diseases, Infections, and Cancers. EMBO Mol. Med..

[25] Lebel E., Nachmias B., Pick M., Gross Even-Zohar N., Gatt M.E. (2022). Understanding the Bioactivity and Prognostic Implication of Commonly Used Surface Antigens in Multiple Myeloma. J. Clin. Med..

[26] Agarbati S., Benfaremo D., Viola N., Paolini C., Svegliati Baroni S., Funaro A., Moroncini G., Malavasi F., Gabrielli A. (2022). Increased Expression of the Ectoenzyme CD38 in Peripheral Blood Plasmablasts and Plasma Cells of Patients with Systemic Sclerosis. Front. Immunol..

[27] Siegel C.H., Sammaritano L.R. (2024). Systemic Lupus Erythematosus: A Review. JAMA.

[28] Adamichou C., Bertsias G. (2017). Flares in Systemic Lupus Erythematosus: Diagnosis, Risk Factors and Preventive Strategies. Mediterr. J. Rheumatol..

[29] Thong B., Olsen N.J. (2016). Systemic Lupus Erythematosus Diagnosis and Management. Rheumatology.

[30] Rodríguez-Bayona B., Ramos-Amaya A., Pérez-Venegas J.J., Rodríguez C., Brieva J.A. (2010). Decreased Frequency and Activated Phenotype of Blood CD27 IgD IgM B Lymphocytes Is a Permanent Abnormality in Systemic Lupus Erythematosus Patients. Arthritis Res. Ther..

[31] Cruciani C., Zen M., Gatto M., Morand E., Doria A. (2023). Assessment of Disease Activity and Damage in SLE: Are We There Yet?. Best Pract. Res. Clin. Rheumatol..

[32] Leffler J., Bengtsson A.A., Blom A.M. (2014). The Complement System in Systemic Lupus Erythematosus: An Update. Ann. Rheum. Dis..

[33] Weinstein A., Alexander R.V., Zack D.J. (2021). A Review of Complement Activation in SLE. Curr. Rheumatol. Rep..

[34] Erdei A., Kovács K.G., Nagy-Baló Z., Lukácsi S., Mácsik-Valent B., Kurucz I., Bajtay Z. (2021). New Aspects in the Regulation of Human B Cell Functions by Complement Receptors CR1, CR2, CR3 and CR4. Immunol. Lett..

[35] Parodis I., Gatto M., Sjöwall C. (2022). B Cells in Systemic Lupus Erythematosus: Targets of New Therapies and Surveillance Tools. Front. Med..

[36] Ceccarelli F., Natalucci F., Olivieri G., Perricone C., Pirone C., Spinelli F.R., Alessandri C., Conti F. (2021). Erosive Arthritis in Systemic Lupus Erythematosus: Not Only Rhupus. Lupus.

[37] Enocsson H., Karlsson J., Li H.-Y., Wu Y., Kushner I., Wetterö J., Sjöwall C. (2021). The Complex Role of C-Reactive Protein in Systemic Lupus Erythematosus. J. Clin. Med..

[38] Kostopoulou M., Mukhtyar C.B., Bertsias G., Boumpas D.T., Fanouriakis A. (2024). Management of Systemic Lupus Erythematosus: A Systematic Literature Review Informing the 2023 Update of the EULAR Recommendations. Ann. Rheum. Dis..

[39] Alduraibi F.K., Tsokos G.C. (2024). Lupus Nephritis Biomarkers: A Critical Review. Int. J. Mol. Sci..

[40] Kitagori K., Yoshifuji H., Oku T., Sasaki C., Miyata H., Mori K.P., Nakajima T., Ohmura K., Kawabata D., Yukawa N. (2016). Cleaved Form of Osteopontin in Urine as a Clinical Marker of Lupus Nephritis. PLoS ONE.

[41] Omer M.H., Shafqat A., Ahmad O., Nadri J., AlKattan K., Yaqinuddin A. (2024). Urinary Biomarkers for Lupus Nephritis: A Systems Biology Approach. J. Clin. Med..

[42] Alenzi F., Ateka-Barrutia O., Ken Cheah C., Khamashta M., Sangle S.R., D’Cruz D.P. (2024). Lupus Nephritis Outcomes after Stopping Immunosuppression. J. Clin. Med..

[43] Imran T.F., Yick F., Verma S., Estiverne C., Ogbonnaya-Odor C., Thiruvarudsothy S., Reddi A.S., Kothari N. (2016). Lupus Nephritis: An Update. Clin. Exp. Nephrol..

[44] Szabó E., Faragó A., Bodor G., Gémes N., Puskás L.G., Kovács L., Szebeni G.J. (2024). Identification of Immune Subsets with Distinct Lectin Binding Signatures Using Multi-Parameter Flow Cytometry: Correlations with Disease Activity in Systemic Lupus Erythematosus. Front. Immunol..

[45] Xiong H., Tang Z., Xu Y., Shi Z., Guo Z., Liu X., Tan G., Ai X., Guo Q. (2022). CD19+CD24highCD27+ B Cell and Interleukin 35 as Potential Biomarkers of Disease Activity in Systemic Lupus Erythematosus Patients. Adv. Rheumatol..

[46] Zhou H., Li B., Li J., Wu T., Jin X., Yuan R., Shi P., Zhou Y., Li L., Yu F. (2019). Dysregulated T Cell Activation and Aberrant Cytokine Expression Profile in Systemic Lupus Erythematosus. Mediat. Inflamm..

[47] Loeser R.F., Arbeeva L., Kelley K., Fodor A.A., Sun S., Ulici V., Longobardi L., Cui Y., Stewart D.A., Sumner S.J. (2022). Association of Increased Serum Lipopolysaccharide, But Not Microbial Dysbiosis, With Obesity-Related Osteoarthritis. Arthritis Rheumatol..

