# Supplementary material for: Circulating B Lymphocyte Subsets in Patients with Systemic Lupus Erythematosus
Source: Medicina (Kaunas). 2024 Dec 2;60(12):1994. doi: 10.3390/medicina60121994 (PMC11727997; doi:10.3390/medicina60121994)
Supplement: Supplementary file 1 [file medicina-60-01994-s001.zip › medicina-3318528-supplementary.pdf]

Online Supplement

Circulating B lymphocyte subsets in patients with systemic lupus erythematosus

Joanna Kosałka-Węgiel, Bogdan Jakieła, Radosław Dziedzic, Mamert Milewski, Andżelika Siwiec-Koźlik, Lech Zaręba, Stanisława Bazan-Socha, Marek Sanak, Jacek Musiał, Mariusz Korkosz

Correspondence to
Joanna Kosałka-Węgiel, MD, PhD
Jagiellonian University Medical College, Department of Rheumatology and Immunology
Jakubowskiego 2
30-688 Kraków, Poland
phone: +48 12 400 31 10
e-mail: joanna.kosalka@uj.edu.pl
ORCID: 0000-0003-1013-2253

Supplementary Table S1. B cell subsets analysis in active systemic lupus erythematosus patients with renal flare and other than renal flare.

Parameter	Active SLE patients with renal flare n = 5	Active SLE patients with non-renal flare n = 7	p-value
CD19+, % of lymphocytes	8.2 (7.1-16.4)	5.7 (5.4-17.8)	0.43
Naive, % of B lymphocytes	60.2 (53.9-73.1)	63.4 (38.3-86.6)	1.00
SM, % of B lymphocytes	14.1 (7.5-26.3)	18.1 (5.5-37.9)	0.91
NSM, % of B lymphocytes	10.0 (4.7-27.8)	6.5 (2.9-9.4)	0.26
DN, % of B lymphocytes	5.0 (0.7-10.0)	5.0 (1.6-10.7)	0.76
Plasmocytes, % of B lymphocytes	0.5 (0.1-1.5)	0.6 (0.1-2.9)	0.93
Plasmablasts, % of B lymphocytes	0.9 (0.3-3.1)	1.8 (0.7-7.0)	0.32
B-trans/reg, % of B lymphocytes	3.6 (2.4-7.6)	3.8 (0.8-5.0)	0.79
B cells per µl			
CD19+, cells/µl	94.8 (40.7-277.5)	88.5 (30.1-137.1)	0.64
Naive, cells/µl	52.7 (19.0-120.8)	39.9 (10.5-126.4)	0.79
SM, cells/µl	10.1 (8.4-13.0)	19.3 (4.1-28.8)	0.61
NSM, cells/µl	7.1 (2.2-59.0)	4.7 (3.0-8.2)	0.76
DN, cells/µl	2.2 (1.1-4.1)	4.1 (2.9-5.7)	0.17
Plasmocytes, cells/µl	0.4 (0.2-0.5)	0.6 (0.2-1.0)	0.53
Plasmablasts, cells/µl	0.8 (0.2-1.8)	1.3 (0.7-3.0)	0.32
B-trans/reg, cells/µl	4.3 (1.4-6.6)	1.0 (0.9-5.2)	0.79

Continuous variables are presented as median with Q1-Q3 ranges. Abbreviations: B trans/reg – transitional/regulatory B cells, CD – cluster of differentiation, DN – double negative, n – number, NSM – non-switched memory, SLE – systemic lupus erythematosus, SM – switched memory.

Supplementary Table S2. B cell subsets analysis in active systemic lupus erythematosus patients with remission and lupus nephritis versus remission without lupus nephritis.

**Parameter**	**Inactive SLE patients with remission and lupus nephritis** n = 10	**Inactive SLE patients with remission without lupus nephritis** n = 13	**p-value**
CD19+, % of lymphocytes	5.6 (4.5-8.9)	6.7 (3.5-11.8)	0.97
Naive, % of B lymphocytes	77.9 (46.1-87.7)	67.5 (50.7-83.1)	0.51
SM, % of B lymphocytes	10.0 (5.5-25.6)	15.7 (8.5-39.0)	0.44
NSM, % of B lymphocytes	6.1 (3.2-9.9)	8.7 (3.4-10.9)	0.44
DN, % of B lymphocytes	2.7 (1.3-4.7)	2.8 (1.5-6.7)	0.73
Plasmocytes, % of B lymphocytes	0.4 (0.2-0.7)	0.2 (0.1-1.1)	0.41
Plasmablasts, % of B lymphocytes	0.5 (0.4-7.6)	1.5 (0.7-2.0)	0.41
B-trans/reg, % of B lymphocytes	2.2 (0.5-3.1)	2.4 (0.9-4.8)	0.38
B cells per µl			
CD19+, cells/µl	119.9 (80.1-149.7)	54.8 (24.6-148.7)	0.20
Naive, cells/µl	73.3 (50.0-130.2)	37.3 (10.4-117.8)	0.20
SM, cells/µl	10.3 (5.4-20.5)	13.4 (4.5-17.4)	0.74
NSM, cells/µl	7.6 (2.0-9.3)	4.8 (1.1-10.1)	0.61
DN, cells/µl	2.0 (1.2-6.6)	2.6 (0.8-3.6)	0.74
Plasmocytes, cells/µl	0.3 (0.1-0.6)	0.2 (0.1-0.7)	0.66
Plasmablasts, cells/µl	0.7 (0.5-2.2)	0.6 (0.4-2.2)	0.50
B-trans/reg, cells/µl	2.3 (0.2-5.4)	1.1 (0.3-4.8)	0.82

Continuous variables are presented as median with Q1-Q3 ranges. Abbreviations: B trans/reg – transitional/regulatory B cells, CD – cluster of differentiation, DN – double negative, n – number, NSM – non-switched memory, SLE – systemic lupus erythematosus, SM – switched memory.

Supplementary Table S3. B cell subsets analysis in inactive systemic lupus erythematosus patients with a flare and without a flare in the follow-up analysis.

Parameter	**Inactive SLE patients with a flare in the follow-up** n = 5	**Inactive SLE patients without a flare in the follow-up** n = 18	p-value
CD19+, % of lymphocytes	4.0 (1.3-14.8)	6.5 (4.3-10.0)	0.39
Naive, % of B lymphocytes	71.3 (31.9-83.4)	73.2 (50.8-85.2)	0.66
SM, % of B lymphocytes	12.8 (5.8-49.9)	13.4 (7.7-24.6)	1.00
NSM, % of B lymphocytes	10.8 (4.1-15.0)	5.1 (3.4-9.7)	0.33
DN, % of B lymphocytes	4.0 (2.1-7.3)	2.5 (1.2-4.5)	0.38
Plasmocytes, % of B lymphocytes	0.3 (0.1-3.4)	0.4 (0.2-0.7)	0.62
Plasmablasts, % of B lymphocytes	1.2 (0.6-11.2)	1.2 (0.5-3.3)	0.68
B-trans/reg, % of B lymphocytes	3.3 (0.7-5.0)	2.0 (0.8-3.4)	0.50
B cells per µl			
CD19+, cells/µl	98.1 (8.1-190.7)	88.5 (48.6-135.7)	0.84
Naive, cells/µl	75.9 (3.2-164.7)	53.2 (33.2-107.8)	0.96
SM, cells/µl	10.3 (3.0-10.3)	15.3 (5.9-18.7)	0.30
NSM, cells/µl	4.0 (0.8-4.0)	6.5 (1.9-8.8)	1.00
DN, cells/µl	3.6 (0.1-3.6)	2.1 (0.9-3.6)	0.77
Plasmocytes, cells/µl	0.2 (0.1-0.2)	0.3 (0.1-0.6)	0.49
Plasmablasts, cells/µl	0.7 (0.3-0.7)	0.7 (0.4-2.8)	0.63
B-trans/reg, cells/µl	3.4 (0.3-8.1)	1.7 (0.3-4.2)	0.82

Continuous variables are presented as median with Q1-Q3 ranges. Abbreviations: B trans/reg – transitional/regulatory B cells, CD – cluster of differentiation, DN – double negative, n – number, NSM – non-switched memory, SLE – systemic lupus erythematosus, SM – switched memory.
